# Scaling laws for ion irradiation effects in iron-based superconductors

**DOI:** 10.1038/s41598-021-84699-4

**Published:** 2021-03-12

**Authors:** Daniele Torsello, Laura Gozzelino, Roberto Gerbaldo, Tsuyoshi Tamegai, Gianluca Ghigo

**Affiliations:** 1 Department of Applied Science and Technology, Politecnico di Torino, 10129 Turin, Italy; 2 Istituto Nazionale di Fisica Nucleare, Sezione di Torino, 10125 Turin, Italy; 3 Department of Applied Physics, The University of Tokyo, Bunkyo-ku, Tokyo, 113-8656 Japan

**Keywords:** Superconducting properties and materials, Characterization and analytical techniques, Scaling laws

## Abstract

We report on ion irradiation experiments performed on compounds belonging to the $\hbox {BaFe}_2\hbox {As}_2$ family, each one involving the partial substitution of an atom of the parent compound (K for Ba, Co for Fe, and P for As), with an optimal composition to maximize the superconducting critical temperature $T_c$. Employed ion beams were 3.5-MeV protons, 250-MeV Au ions, and 1.2-GeV Pb ions, but additional data from literature are also considered, thus covering a wide range of ions and energies. Microwave characterization based on the use of a coplanar waveguide resonator allowed us to investigate the irradiation-induced $T_c$ degradation, as well as the increase of normal state resistivity and London penetration depth. The damage was quantified in terms of displacements per atom (dpa). From this broad and comprehensive set of experimental data, clear scaling laws emerge, valid in the range of *moderate* irradiation-induced disorder (dpa up to 5 $\times$ 10 $^{-3}$ were investigated). In these conditions, linear trends with dpa were found for all the modification rates, while a power law dependence on the ion energy was found for heavy-ion irradiation. All these scaling laws are reported and discussed throughout the paper.

## Introduction

Creation of defects in superconductors via ion irradiation is one of the most promising ways to optimize their properties for applications^1–3^ and the most efficient tool to study the disorder dependence of their fundamental properties^4–7^.

Irradiation-induced defects act as pinning centers, enhancing vortex pinning and critical current density ($J_c$). By selecting the irradiation conditions, such as ion species and energy, it is possible to create defects that range from point-like to columnar. All these defects are effective in increasing vortex pinning and $J_c$^8^, and the details of pinning strength and anisotropy vary depending on the morphology of the created defects^9,10^. This approach can be used also on coated conductors and its scalability is promising for industrial applications^3,11,12^. Both uniform distributions of defects and pre-determined patterns can be obtained using defocused beams and collimated beams or masks, respectively^13–15^. However, defects also act as scattering centers for charge carriers, resulting in a decrease of the critical temperature of the material, $T_c$, and in an increase of the normal state resistivity, $\rho$, and of the London penetration depth, $\lambda$. This often undesired effect has been a subject of fundamental studies aimed at the determination of the pairing state of newly discovered superconductors^16–18^. For the sake of completeness, it should be mentioned that in some cases also $T_c$ enhancement as a result of irradiation was reported^19,20^. This has usually been discussed in terms of indirect effects: either structural modifications (increase of strain) of the superconducting material due to the irradiation induced modification of the substrate in thin films^20^ or by suppression of competing orders^19^.

Due to the virtually infinite range of possible ion-energy-target combinations, the comparison of irradiation results is often challenging. Therefore, studies focused on specific classes of superconductors and involving a large variety of ions and energies are needed, aimed at deducing scaling laws that could be useful to guide further irradiation experiments and possibly to design applications. In fact, the ability to modify and control key properties of materials opens the way to a number of possible applications, ranging from coated conductors with engineered critical current^3^ to devices (e.g. for vortex guidance)^21^, when modifications can be controlled locally^13,22^. In this framework, ion irradiation can play a role - in preparatory studies or in industrial processes - but the possibility of reducing the number of ion-energy combination to be investigated experimentally for a specific application through quick and easy computer simulations and rule-of thumb relations would represent a great help and would greatly improve efficiency.

In this work, we focus on the Ba-122 family of the iron-based superconductors (IBSs), with different substitutions implying different charge dopings, and we analyze the $T_c$ degradation rate and the increase of $\rho$ and $\lambda$ due to exposition to different ion beams, ranging from MeV-protons to GeV-heavy-ions. Samples were characterized before and after irradiation by a microwave technique^23–25^ (described in the Methods section) allowing us to monitor modifications in $T_c$, $\rho$, and $\lambda$. We investigate the existence of scaling relations between degradation rates and irradiation-induced damage, calculated in terms of displacements per atom (dpa), and particle energy, as well as between degradation rates and pristine critical temperature for different materials exposed to the same irradiation conditions.

## Results

**Figure 1:**
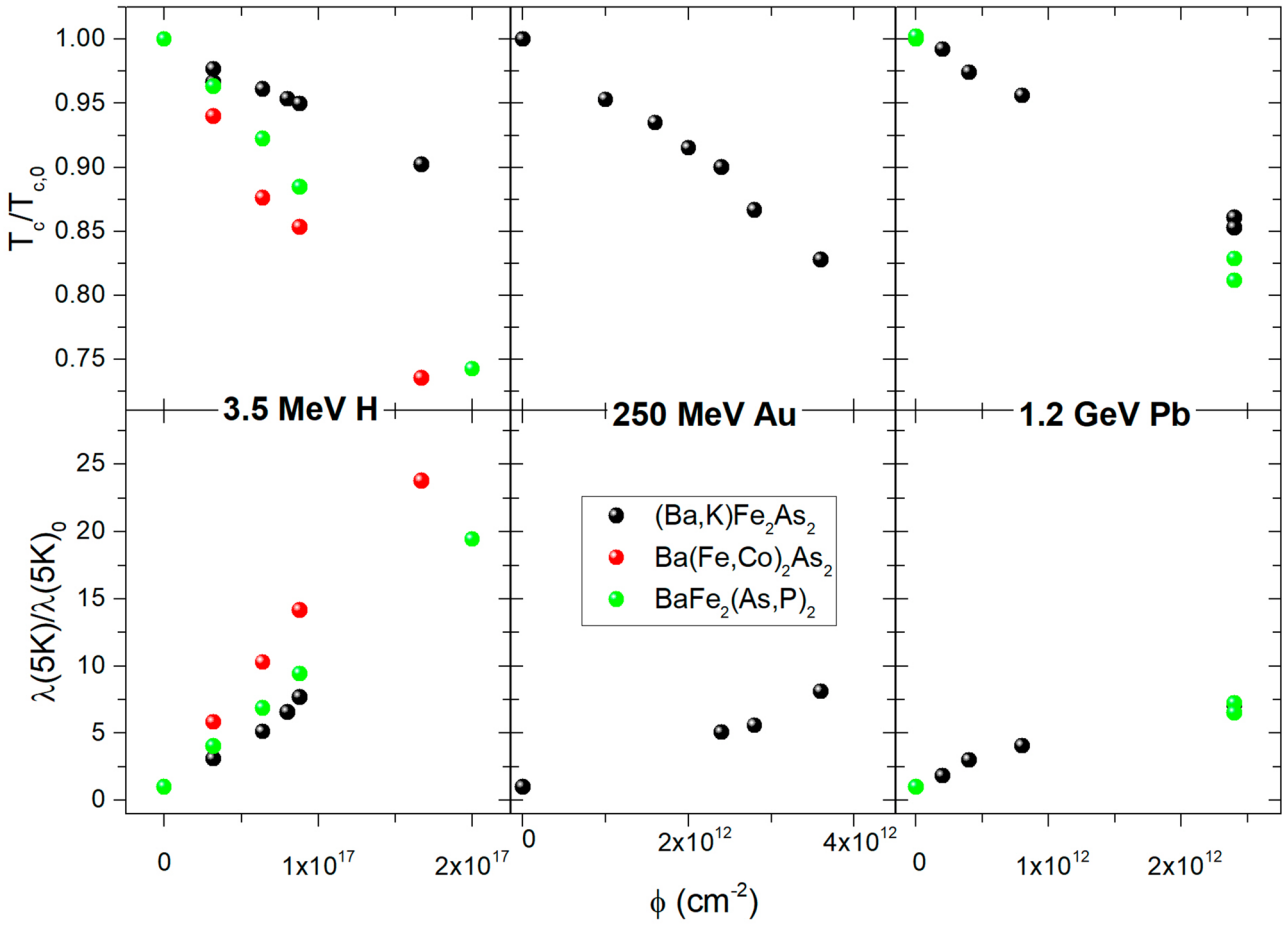
Critical temperature degradation (top panels) and low temperature London penetration depth increase (bottom panels) expressed as the ratio between the values for a certain fluence and for the pristine sample ($T_c/T_{c,0}$ and $\lambda$(5K)/$\lambda$(5K)$_{0}$), as a function of irradiation fluence, for 3.5 MeV proton irradiation on the left, 250 MeV Au ion irradiation at the center, and 1.2 GeV Pb ion irradiation on the right. Absolute values for the pristine samples are reported in the Methods section.

Figure 1 shows the increase of the low temperature value of the London penetration depth, $\lambda$(5K), and the critical temperature degradation of the materials as a function of the irradiation fluence for the three types of irradiation (and resulting defects) described in the Methods section, employing 3.5-MeV protons, 250-MeV Au ions, and 1.2-GeV Pb ions. $T_c$ degradation and $\lambda$(5K) increase are expressed as the ratio of their value after irradiation with a certain dose, with respect to the value of the same sample in the pristine state, $T_{c,0}$ and $\lambda$(5K)$_0$. Absolute values for the pristine samples are reported in the Methods section.

It is evident that similar $T_c$ degradation values are observed for fluences of different orders of magnitude when different combinations of ion and energy are considered. This fact poses strong limitations to the possibility of comparing the outcomes of different irradiation experiments. Therefore, the most straightforward way to compare different irradiation effects is to compute the amount of disorder introduced in the system through the irradiation process. As discussed in details in the Methods section, a good, commonly used, and easy to compute, parameter to quantify the amount of defects is the number of displacements per atom, dpa.

**Figure 2:**
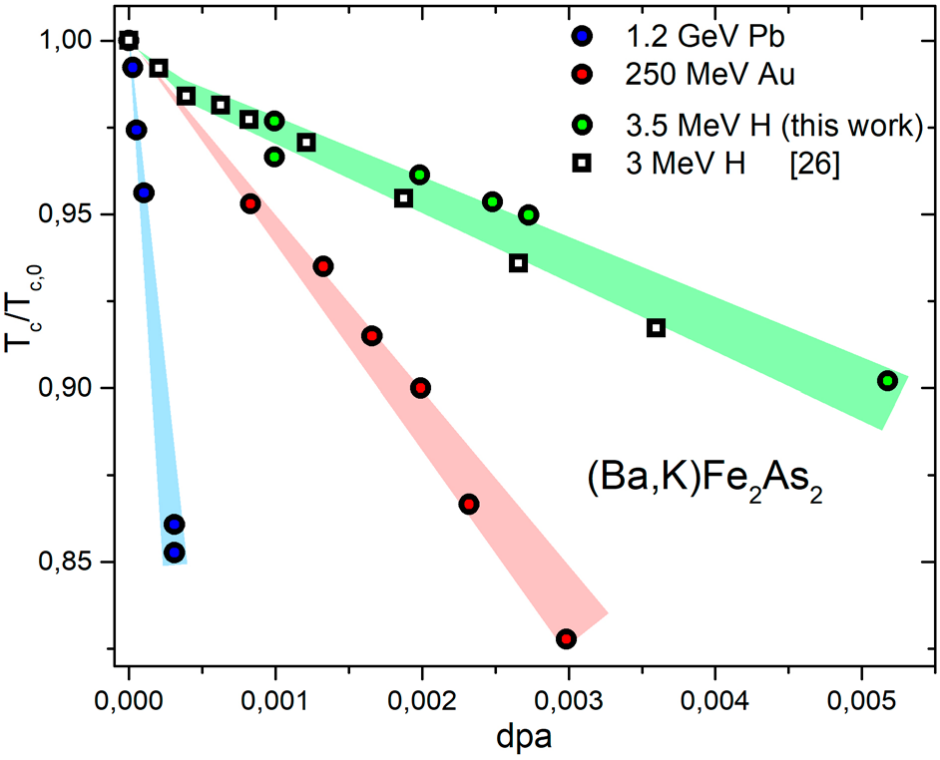
Critical temperature degradation as a function of the amount of introduced defects in terms of dpa for different ion-energy combinations on the same type of IBS (optimally doped $$\hbox {Ba}_{1-x}$$$$\hbox {K}_{x}$$$$\hbox {Fe}_{2}$$$$\hbox {As}_{2}$$). Data for 3 MeV H irradiation (white squares) are taken from Ref.^26^ and obtained by low temperature irradiation and in-situ dc resistivity measurement.

**Figure 3:**
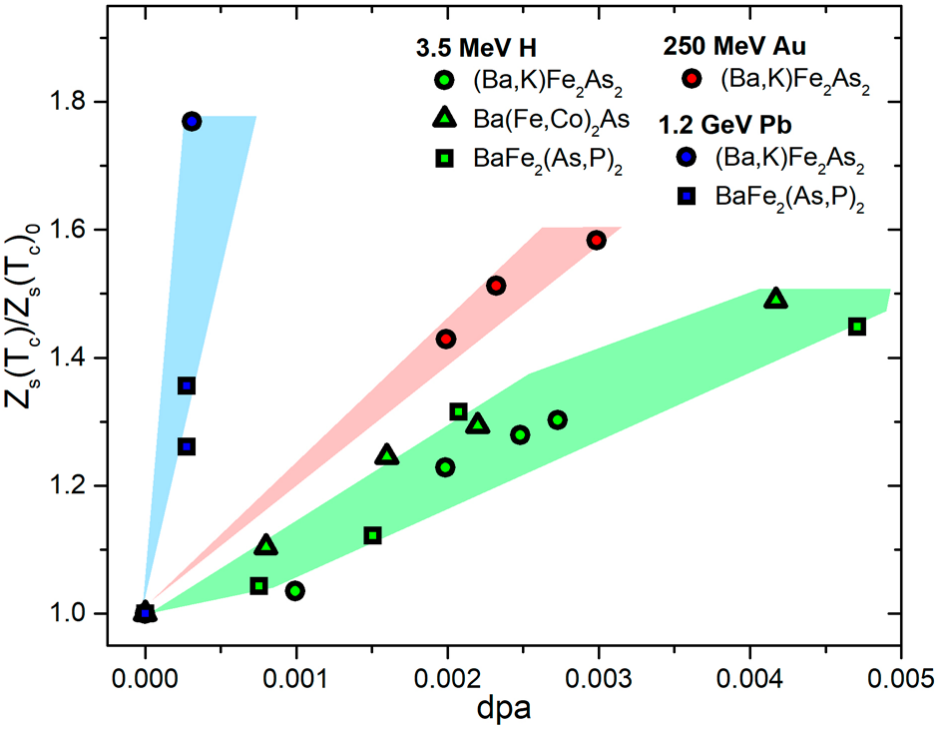
Surface impedance (at $T_c$) variation as a function of the amount of introduced defects in terms of dpa for different irradiation ion-energy combinations.

**Figure 4:**
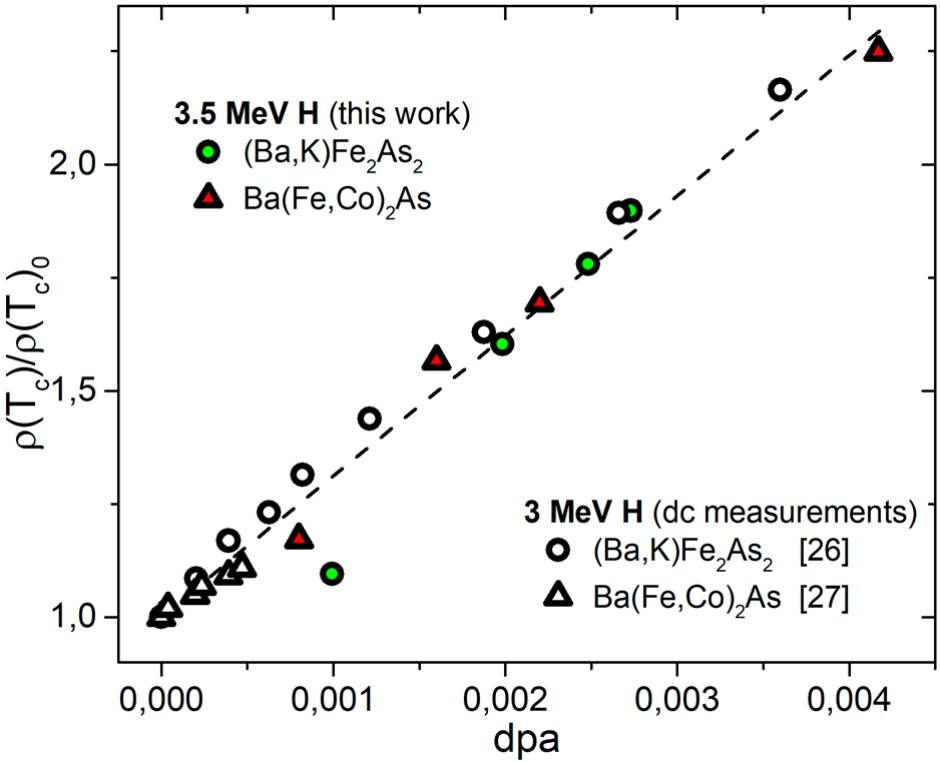
Resistivity (at $$T_c$$) increase as a function of the amount of introduced defects in terms of dpa for proton irradiated optimally doped $$\hbox {Ba}_{1-x}$$$$\hbox {K}_{x}$$$$\hbox {Fe}_{2}$$$$\hbox {As}_{2}$$ and Ba($$\hbox {Fe}_{1-x}$$$$\hbox {Co}_x$$)$$_{2}$$$$\hbox {As}_{2}$$. Data for 3 MeV H irradiation (white symbols) are taken from Refs.^26,27^ and obtained by low temperature irradiation and in-situ dc resistivity measurement.

Figure 2 shows the $$T_c$$ degradation of $$\hbox {Ba}_{1-x}$$$$\hbox {K}_{x}$$$$\hbox {Fe}_{2}$$$$\hbox {As}_{2}$$ as a function of the dpa introduced via ion irradiation. It is evident that in all cases the behavior is qualitatively similar (linear decrease) but quantitatively very different for ions and energies that create different types of defects.

**Figure 5:**
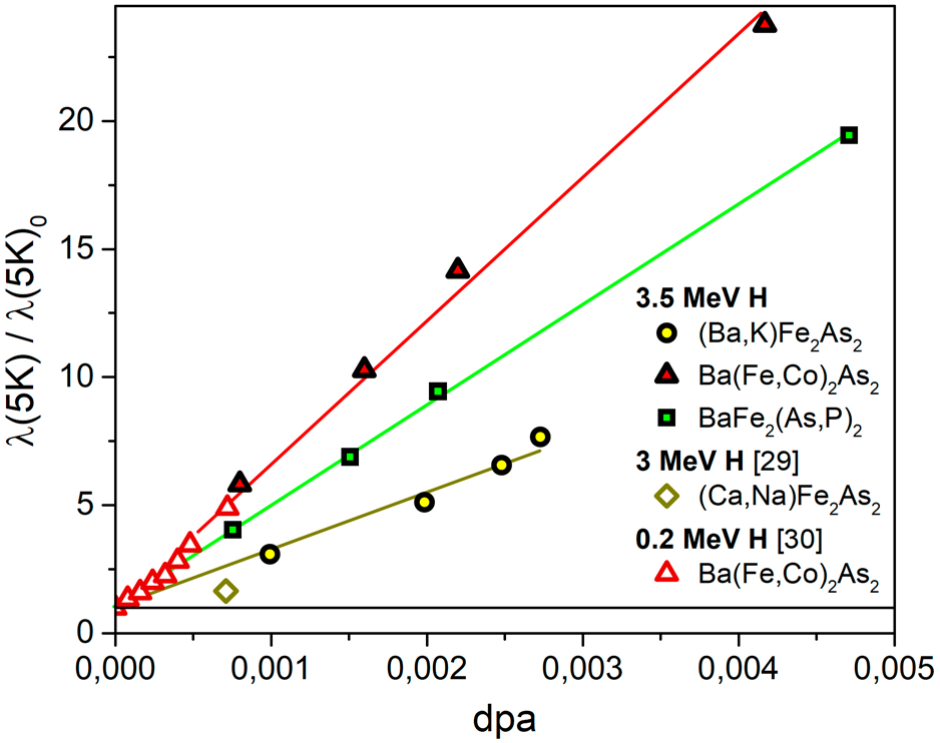
Low temperature London penetration depth of different IBS samples irradiated with 3.5-MeV protons, normalized to the value for the pristine one, as a function of dpa, fitted by a linear trend. Data from this work are compared to data from literature^29,30^.

The same type of dependency is shown in Figs. 3 and 4 for the variation of the surface impedance value and of the resistivity at the critical temperature. Figure 5 shows instead the variation of the low temperature London penetration depth.

For the cases of $T_c$ degradation and resistivity increase, it was possible to compare our data with similar data from the literature^26,27^ obtained by low temperature 3-MeV proton irradiation and dc-resistivity measurements. The defects created by 3-MeV and 3.5-MeV protons are expected to be identical (see the Methods section for further details) and their amounts can be well estimated through the dpa value. Therefore, the comparison of these two datasets (Figs. 2 and 4) allows us to discuss differences between room temperature and low temperature proton irradiation. The two processes seem to yield a very similar $T_c$ degradation and an almost identical resistivity increase for both K-doped and Co-doped 122 IBSs. The slight difference visible in the $T_c$ degradation, could be due to the different measurement technique (dc vs. microwave) and/or to a smaller recombination rate of created defects at low temperatures. However, the effect is extremely small. The fact that this difference does not show up in the resistivity could be due to a greater sensitivity to scattering induced by disorder of superconducting properties with respect to normal state properties.

Despite the penetration depth in irradiated crystals is more seldom measured, some data is indeed available. Therefore, in Fig. 5 we compare our data, concerning the modification of penetration depth due to irradiation, with other findings from literature. What immediately captures the attention is the huge modification of $$\lambda$$ despite the relatively limited degradation of $$T_c$$. This is not new: a similar behavior was observed in substituted and irradiated $$\hbox {YBa}_2$$$$\hbox {Cu}_3$$$$\hbox {O}_{7-\delta }$$ films^28^, and even higher modifications of $$\lambda$$ under irradiation was reported for IBSs. In this last case, very few data are available for Ba122 or similar systems^29,30^. They are reported in Fig. 5 for a comparison with our data, as a function of dpa we calculated on the basis of the information reported in the original papers. Data for the same compound $$\hbox {(Ba(Fe,Co)}_2$$$$\hbox {As}_2$$) nicely agree, despite the different proton energy and sample morphology (single crystals in our case, thin films in Ref.^30^). Such a huge effect on $$\lambda$$ with a significantly weaker effect on $$T_c$$ was explained in terms of Bogoliubov–de Gennes formalism for systems with short coherence lengths, where the order parameter is strongly suppressed in the vicinity of defects but mostly unaffected elsewhere^29,31^. Finally, we note that a similar behavior can be reproduced within a multiband Eliashberg framework in presence of disorder, as we did for proton irradiated Ba122 in Ref.^17^.

## Discussion

**Figure 6:**
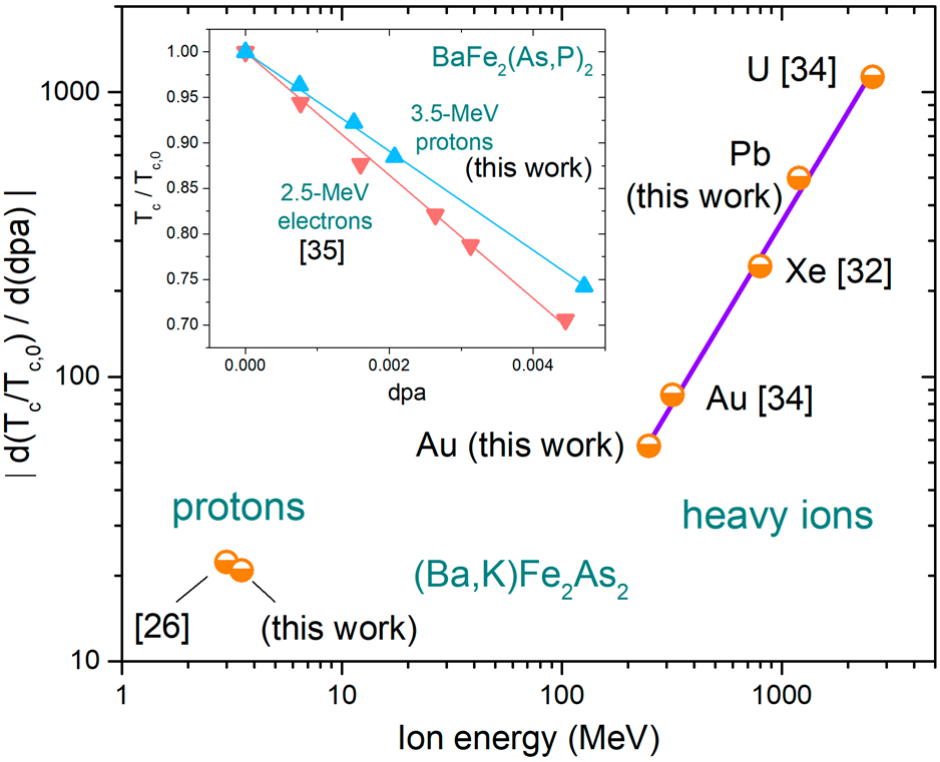
Absolute value of the critical temperature degradation rate, $$|d(T_c/T_{c,0})/d(dpa)|$$, of (Ba,K)$$\hbox {Fe}_2$$$$\hbox {As}_2$$ crystals as a function of the energy of the ion used for irradiation. H, Au, Xe and U irradiation data were also taken from^26,32–34^. Data for heavy ions are fitted by a linear dependence in the log–log scales, leading to the scaling relation reported below in Table 1. The inset shows a comparison of $$T_c$$ degradation for $$\hbox {BaFe}_2$$(As,P)$$_2$$ crystals irradiated with 3.5-MeV protons (this work) and with 2.5-MeV electrons (from^35^), fitted by a linear trend.

With the aim of finding common behaviors, trends, and scaling laws, it is interesting to compare the slopes of the linear relations of the $$T_c$$ degradation for the different cases. Two aspects can be investigated, one related to the effect of different ion irradiations on the superconducting properties, and one related to the intrinsic radiation hardness of a specific material. To discuss the former, we compare irradiations performed with several ions and on a wide energy span, on the same material. For the latter, we investigate the response of differently doped materials to the same irradiation conditions. In Fig. 6, $$T_c$$ degradation rates are presented for $$\hbox {Ba}_{1-x}$$$$\hbox {K}_{x}$$$$\hbox {Fe}_{2}$$$$\hbox {As}_{2}$$ as a function of the energy of the ion used for irradiation. In addition to the data extracted from Fig. 2 obtained from our experiments, also data from literature^26,27,32–34^ are shown.

**Figure 7:**
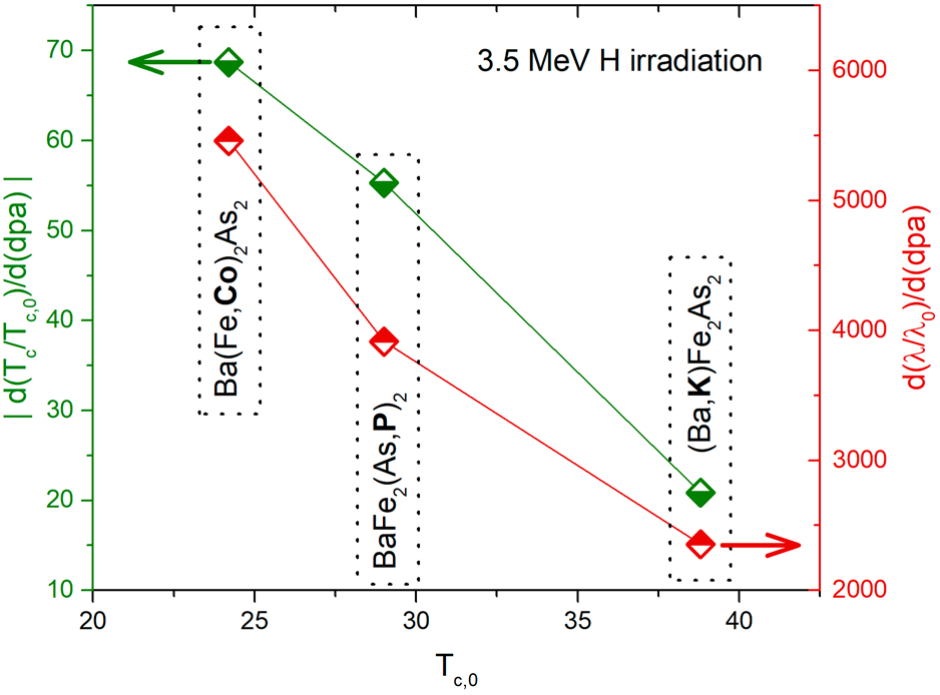
Scaling relations of the absolute value of the critical temperature degradation rate, $$|d(T_c/T_{c,0})/d(dpa)|$$, and London penetration depth increase rate, $$d(\lambda$$(5K)/$$\lambda$$(5K)$$_0)/d(dpa)$$, with the critical temperature of the pristine sample, representative of the strength of the superconducting state.

Generally, it can be noticed that the higher the energy is, the faster is the degradation of the critical temperature with damage (dpa). This could be due to a different efficiency in defect production at different energies or to the fact that dpa alone does not completely account for all the structural consequences of ion irradiation. In fact, for high-energy heavy ions an important mechanism which is not accounted for by dpa is the deposition of energy via ionization, causing deformation/melting of the lattice along the ion track, when a given threshold of the electronic stopping power is overcome. This very well known mechanism is responsible for the production of linearly correlated defects and continuous columnar tracks in a variety of materials, including IBSs^8,34,36,37^. Aiming at addressing whether these could be the defects relevant for $T_c$ degradation, we searched for a scaling law of $T_c/T_{c,0}$ vs. ionization energy, but no clear trend was found. Thus, we conclude that, although columnar defects are decisive to control other important parameters such as critical current and irreversibility field, they are not relevant in the degradation of $T_c$, since they are not as efficient scattering centers as smaller defects are.

On a closer look, Fig. 6 reveals a clear scaling law for all the data involving heavy ions, both from this work and from literature, if $T_c$-degradation slopes as a function of dpa are considered (on the contrary, attempts to correlate $T_c$ degradation to the electronic stopping power $S_e$ were not successful). The existence of this power-law trend (a straight line, in the adopted log–log scales), indicates that defects that are accounted for through dpa, as those stemming from the nuclear stopping power, are efficient scattering centers. These defects are typically nano-sized cascades that are generally quite homogeneously distributed throughout the sample, also far from columnar tracks, when present. This is due to the fact that a primary knock-on atom generated along the track will have enough energy to further travel in the material. Therefore, one can assume that generally the whole material outside the columnar defects is modified by irradiation. For the sake of completeness, a similar process in heavy-ion irradiated IBSs could be ascribed to secondary electrons, but the estimate of the amount of defects in this case is rather complex and deserves to be treated in a separate work.

A different behavior is shown by proton-irradiated samples, with a $$T_c$$ degradation rate out of the heavy-ion trend. This may reflect the different size and morphology of the defects, point-like and small cascades in this case. To validate this hypothesis, we compare in the inset of Fig. 6 the effects of 3.5-MeV protons with the effects of 2.5-MeV electrons (from^35^), which are well known to produce point-like defects. Unfortunately, in literature there is a lack of data about electron irradiation of (Ba,K)$$\hbox {Fe}_2$$$$\hbox {As}_2$$ with a robust statistics, therefore we made the comparison with $$\hbox {BaFe}_2$$(As,P)$$_2$$ crystals. It turns out that the $$T_c$$ degradation rates of proton and electron irradiated samples is quite similar (slopes within $$\sim$$20%), with the differences that, besides the different size of defects, could also be ascribed to the irradiation temperature (20 K in the case of electrons against room temperature for protons) and the experimental probe (dc vs. microwave frequencies) as already discussed for proton irradiation in the previous chapter.

Another interesting aspect, is the relevance of the properties of the pristine material for its response to the introduction of defects. To investigate this, we plot in Fig. 7 the $$T_c$$ degradation rate and London penetration depth increase of different optimally doped compounds of the $$\hbox {BaFe}_2$$$$\hbox {As}_2$$ family exposed to 3.5 MeV proton irradiation, as a function of their pristine critical temperature. By only considering members of the same family, all at optimal doping level, we reduce the number of differences between the samples. Also in this case we find a clear trend: the system with higher $$T_{c,0}$$ is less subject to critical temperature degradation and $$\lambda$$ increase. This means that not only the superconducting order is stronger in the pristine state, but it is also more resilient to the introduction of disorder.

**Table 1:** The table summarizes the scaling laws discussed throughout the paper. Each of them deals with the physical quantity listed in the first column, and is reported in the second column as the ratio between the quantity for the irradiated samples and the value for the pristine material (indicated by the subscript 0). These ratios are expressed as a function of disorder (*dpa*, displacements per atom) and ion energy (*E*, MeV). In the third column the used ions and energies are listed, and in the forth one the investigated range of *dpa* is reported. $$c_i$$, with $$i=1, 2, 3, 4$$, and $$\beta$$ are fitting parameters, whose values are reported in the fifth column for each compound (K=$$\hbox {Ba}_{1-x}$$$$\hbox {K}_{x}$$$$\hbox {Fe}_{2}$$$$\hbox {As}_{2}$$, P=$$\hbox {BaFe}_{2}$$$$\hbox {As}_{1-x}$$ $$\hbox {P}_x$$)$$_{2}$$, and Co=Ba($$\hbox {Fe}_{1-x}$$$$\hbox {Co}_x$$)$$_{2}$$$$\hbox {As}_{2}$$). Finally, the last column shows the sources of the data used to deduce the reported scaling laws.

Physical quantity ratio	Scaling law	Ion/energy	*dpa* range	Doping/parameters	Source
Normal state resistivity	$\frac{\rho (T_c)}{\rho (T_c)_0}=1+c_1\cdot (dpa)$	H 3–3.5 MeV	0–0.005	K, P, Co: $c_1=310$	This work, ^26,27^
Penetration depth	$\frac{\lambda (5K)}{\lambda (5K)_0}=1+c_2\cdot (dpa)$	H 3.5 MeV	0–0.005	K: $c_2=2300$ P: $c_2=4000$ Co: $c_2=5600$	This work
Critical temperature	$\frac{T_c}{T_{c,0}}=1-c_3\cdot (dpa)$	H 3.5 MeV	0–0.005	K: $c_3=19$ P: $c_3=55$ Co: $c_3=66$	This work
Critical temperature	$\frac{T_c}{T_{c,0}}=1-c_4\cdot (E/E_0)^\beta \cdot (dpa)$	Heavy ions 250–2600 MeV	0–0.003	K: $c_4=0.052$ $\beta =1.3, E_0=1 \text{ MeV}$	This work, ^26,32–34^

## Conclusions

In this work, we reported on ion irradiation experiments of $$\hbox {BaFe}_2$$$$\hbox {As}_2$$ single crystals, with various substitutions (K for Ba, Co for Fe, and P for As) at the optimal doping level. These compounds fully represent the Ba122 family, since the different substitutions induce chemical pressure, hole and electron doping, and are operated in and out of FeAs planes (that are the main contributors to the material transport properties).

We tried to gain a wide and comprehensive picture of the effects of different kinds of ion irradiation by using a variety of particles/energies, namely 3.5-MeV protons, 250-MeV Au ions, and 1.2-GeV Pb ions, and also considering further data from literature.

Microwave characterization allowed us to investigate the irradiation-induced critical temperature degradation and the increases of normal state resistivity and penetration depth. These quantities were investigated as a function of damage, quantified by the calculated displacements per atom (dpa). Interestingly, datasets obtained by different techniques (*i.e.* microwave and dc-transport) and with different irradiation conditions (room temperature and cryogenic temperature) appear to be highly comparable, indicating that defects recombination and probe frequency play a minor role.

From this broad and comprehensive sets of experimental data, clear scaling laws emerged, which are summarized in Table 1. These laws must be considered within their limits of validity, i.e. in the range of *moderate* irradiation-induced disorder (here dpa up to 5 $\times$ 10 $^{-3}$ were investigated). In these conditions, linear trends were found for all the modification rates. The slopes, useful for a first design of irradiation experiments, are resumed in the Table as $c_i$ parameters.

The analysis also clarified that the defects relevant for $T_c$ degradation in heavy-ion irradiation experiments are those originated mainly by interaction with the nuclei, rather than linear defects created by ionization, although the latter are crucial to control other important parameters such as critical current density and irreversibility field. This results in a clear power-law scaling as a function of the ion energy if the $T_c$-degradation slope with dpa is considered.

Moreover, proton-induced effects, not matching this trend, show a similarity with electron irradiation, known to cause mainly point-like defects.

Overall, the results obtained by this wide analysis represent a sort of guide, useful for preliminary planning of irradiation experiments that can be aimed to fundamental studies, as well as to materials engineering strategies in view of specific applications.

## Methods

### Crystals preparation

Optimally doped single crystals of $$\hbox {Ba}_{1-x}$$$$\hbox {K}_{x}$$$$\hbox {Fe}_{2}$$$$\hbox {As}_{2}$$, Ba($$\hbox {Fe}_{1-x}$$$$\hbox {Co}_x$$)$$_{2}$$$$\hbox {As}_{2}$$, and $$\hbox {BaFe}_{2}$$($$\hbox {As}_{1-x}$$$$\hbox {P}_x$$)$$_{2}$$, with an analyzed doping level of $$x=$$0.42, 0.075, and 0.33, respectively, were grown by the FeAs self-flux method^26,27,38,39^. All the investigated crystals were cleaved and reduced to the form of thin plates with thickness of about 10 $$\mu$$m, in the direction of the *c*-axis of the crystals, more than 10 times smaller than width and length.

### CPWR measurements

The critical temperature, surface impedance and London penetration depth were measured by means of a coplanar waveguide resonator (CPWR) technique that has already been applied to study other IBS crystals^40–43^. The resonator consists of an $$\hbox {YBa}_2$$$$\text{Cu}_3$$$$\text{O}_{7-x}$$ coplanar waveguide to which the sample is coupled. The whole resonance curve is recorded with a vector network analyzer, making it possible to track resonant frequency shifts and variations of the unloaded quality factor. This procedure gives access to the absolute value of the penetration depth and its full temperature dependence, from $$\sim 5$$ K to $$T_c$$^24^, after a calibration procedure has been performed (full details in^23^). Here, the critical temperature is defined as the temperature at which the penetration depth diverges. The most significant properties in the characterization of a material that can be extracted by means of this analysis, and that are discussed in this work are $$T_{c}$$ and $$\lambda$$(5K) (whose inverse squared is proportional to the superfluid density, representative of the density of Cooper pairs in the material^44^). The values of these quantities for the unirradiated crystals are summarized in Table 2.

**Table 2:** Critical temperature and low-temperature London penetration depth for the as-grown crystals, implantation depth (ion range) for each employed energy and ion combinations.

Compound	$T_{c,0}$ (K)	$\lambda$(5K)$_0$ (nm)	3.5 MeV H ion range ($\mu$m)	250 MeV Au ion range ($\mu$m)	1.2 GeV Pb ion range ($\mu$m)
$$\hbox {Ba}_{1-x}$$$$\hbox {K}_{x}$$$$\hbox {Fe}_{2}$$$$\hbox {As}_{2}$$	38.7	197	69	15	39
Ba($$\hbox {Fe}_{1-x}$$$$\hbox {Co}_x$$)$$_{2}$$$$\hbox {As}_{2}$$	24.2	165	70	15	40
$$\hbox {BaFe}_{2}$$($$\hbox {As}_{1-x}$$$$\hbox {P}_x$$)$$_{2}$$	29.0	160	74	16	41

### Ion irradiation

All ion irradiations were performed at the Legnaro National Laboratories of the Italian National Institute for Nuclear Physics (INFN), employing different accelerator facilities for the different ion-energy combinations. At the CN accelerator we used 3.5 MeV protons that produce point-like defects and small clusters in the target material through elastic scattering with the nuclei that compose the crystal lattice. The Tandem-XTU, and the Piave-Alpi facilities allowed us to perform irradiations with heavier ions at higher energies (250 MeV Au and 1.2 Gev Pb respectively) that, in addition to interaction with the nuclei, are characterized by additional production of defects coming from ionization. This process creates linearly correlated defects that range from discontinuous tracks (as those produced by 250 MeV Au ions) to continuous columnar defects (resulting from 1.2 GeV Pb irradiation). The ionization process might also generate energetic electrons that could contribute to the formation of a homogeneous distribution of point defects throughout the whole sample. All irradiations were performed at room temperature, with the beam parallel to the *c*-axis of the crystals, and keeping an ion flux smaller than $9\cdot 10^{11}\text{cm}^{-2}\text{s}^{-1}$ for protons and $1.8\cdot 10^{8}\text{cm}^{-2}\text{s}^{-1}$ for heavy ions to minimize heating effects^26^.

### Calculation of irradiation-induced damage

The range of ions into the material, the nuclear and electronic stopping powers, and the distribution of produced defects were estimated for each case by Monte Carlo simulations performed with the PHITS^45^ and SRIM^46^ codes. Implantation depths (ion ranges) are reported in Table 2, while typical values of the electronic stopping power per impinging particle, $S_e$, are about 33 eV/nm for 3.5 MeV H irradiation, $2.1\times 10^{4}$ eV/nm for 250 MeV Au irradiation, and $3.9\times 10^{4}$ eV/nm for 1.2 GeV Pb irradiation. Then, with the aim of evaluating the density and distribution of displacement defects, we calculated the damage starting from the nuclear stopping power, $S_n$, within the Kinchin–Pease approach and using a threshold displacement energy of 25 eV for all atomic species. This approach is an approximated analytical model that allows one to obtain a quick evaluation of the total displacements per atom (dpa) produced in a cascade initiated by the projectile particle, by estimating the knock out energy of the first atom and disregarding the spatial distribution of the cascade.

The thickness of all investigated samples needs to be smaller than the obtained range value, to ensure that ion implantation is avoided and that a rather homogeneous distribution of defects is achieved^27^. The thickness of studied samples was smaller than 1/5, 1/2 and 1/4 of the implantation depth for H, Au, and Pb irradiation, respectively. This results in a calculated inhomogeneity of the expected dpa along the thickness < 10%, 25%, and 10%, respectively. These values should be considered as the worst case scenario, since defect diffusion is not taken into account, and when multiple irradiations are performed on the same sample, the surface facing the beam is flipped each time. CPWR measurements were carried out before and after each irradiation session. Once the dpa distribution as a function of depth inside the material is calculated, it is possible to estimate the dpa value for a specific sample as an average over its thickness. This value is useful to compare the effects of different types of defects on the properties of a material and allows one to look for scaling relations and common behaviors.

In addition to the estimate of dpa produced by ions, also the dpa resulting from electron irradiation can be computed and their effects can be compared. In the case of 2.5 MeV electron irradiation, the overall dpa can simply be computed by multiplying the electron fluence by the displacement cross section for each atom, and summing atomic dpa contributions weighted on their stoichiometric coefficients^47^. The displacement cross section is composed of the cross section for primary knock-on atoms (derived by McKinley and Feshbach^48^) and that for the (very small) cascade that each primary atom could produce. This last factor is computed through the mean recoil energy transferred by 2.5 MeV electrons to a target atom^49^.
